# Complete chloroplast genome of *Achyranthes bidentata* Blume

**DOI:** 10.1080/23802359.2019.1674219

**Published:** 2019-10-09

**Authors:** Inkyu Park, Sungyu Yang, Wook Jin Kim, Byeong Cheol Moon

**Affiliations:** Herbal Medicine Resources Research Center, Korea Institute of Oriental Medicine, Naju, Republic of Korea

**Keywords:** *Achyranthes bidentata* Blume, medicinal plant, chloroplast genome, Amaranthaceae

## Abstract

*Achyranthes bidentata* Blume is an important herbal medicine. However, little genetic information about *Achyranthes* is available. To facilitate species identification, we sequenced its complete chloroplast genome using the Illumina MiSeq platform. The data show that the chloroplast genome of *A. bidentata* is 151,441 bp in size and comprises an 83,890 bp large single-copy region, a 17,261 bp small single-copy region, and two inverted repeat (IR) regions, IRa and IRb (each 25,145 bp). The genome contains 112 unique genes, including 79 protein-coding genes, 4 ribosomal RNAs genes, and 30 transfer RNAs genes. Phylogenetic analysis revealed that *A. bidentata* is most closely related to *Alternanthera philoxeroides*.

*Achyranthes bidentata* Blume, a member of the Rosaceae family, is widely distributed in East Asia. Dried roots of *A. bidentata*, called Achyranthis Radix, are used as a Korean traditional herbal medicine to treat anti-arthritic, anti-allergic, anti-diuretic, and other diseases (Korea Institute of Oriental Medicine (KIOM) 2019). Unfortunately, other similar plant species are mixed with *A. bidentata*, a valuable herbal medicine, and indiscriminately misused in Korean herbal markets (Moon et al. 2017). Thus, to accurately identify *A. bidentata* and discriminate it from other similar species, we sequenced its complete chloroplast genome.

Fresh leaves of *A. bidentata* were collected from its native habitat in Korea (35°01′28.9″ N and 127°10′22.2″ E). Specimens were labelled with unique identification numbers and registered in the Korean Herbarium of Standard Herbal Resources (Index herbarium code KIOM) at the Korea Institute of Oriental Medicine (KIOM), with voucher number KIOM201401009429. Genomic DNA was extracted from leaf samples using a DNeasy Plant Maxi Kit (Qiagen, Valencia, CA). An Illumina paired-end library was constructed and sequenced using the MiSeq platform (Illumina Inc., San Diego, CA). The complete chloroplast genome of *A. bidentata* was deposited in the GenBank database of the National Centre for Biotechnology Information under accession number MN255842.

Illumina sequencing of *A. bidentata* yielded 1.418 Gb of high-quality paired-end reads. The chloroplast genome sequence contigs of *A. bidentata* were assembled *de novo* from low-coverage whole-genome sequences (Kim et al. 2015). The complete chloroplast genome of *A. bidentata* comprised 151,441 bp. The chloroplast genome showed a typical quadripartite structure comprising a large single-copy (LSC) region of 83,890 bp, a small single-copy (SSC) region of 17,261 bp, and two inverted repeat (IR) regions, IRa and IRb (each 25,145 bp). The GC content of the chloroplast genome was 36.47%, with the IR regions showing a higher GC content (42.51%) than the LSC (34.18%) and SSC (30%) regions. These data indicate that the chloroplast genome of *A. bidentata* is AT-rich, which is consistent with the chloroplast genomes of other plant species (Park et al. 2017). The chloroplast genome of *A. bidentata* harboured 112 unique genes, including 79 protein-coding genes, 30 genes encoding transfer RNA (tRNAs), and 4 genes encoding ribosomal RNAs (rRNAs). Of the 112 genes, 17 were duplicated in the IR regions and 16 contained introns. Among the genes containing introns, 14 contained a single intron and 2 (*ycf3* and *clpP*) harboured two introns.

To investigate the phylogenetic relationship between *A. bidentata* and other plant species, we aligned the nucleotide sequences of 64 protein-coding genes of *A. bidentata* with those of homologues from 10 other taxa, spanning a total length of 43,976 bp. The phylogenetic tree constructed using the maximum-likelihood (ML) method contained nine nodes, each with bootstrap values of 100% (Figure 1), indicating a strong phylogenetic relationship with the Amaranthaceae family. Additionally, the phylogenetic tree revealed that *A. bidentata* formed a monophyletic group with *Alternanthera philoxeroides* within Amaranthaceae, with bootstrap support values of 100% (Figure 1).

**Figure 1.**
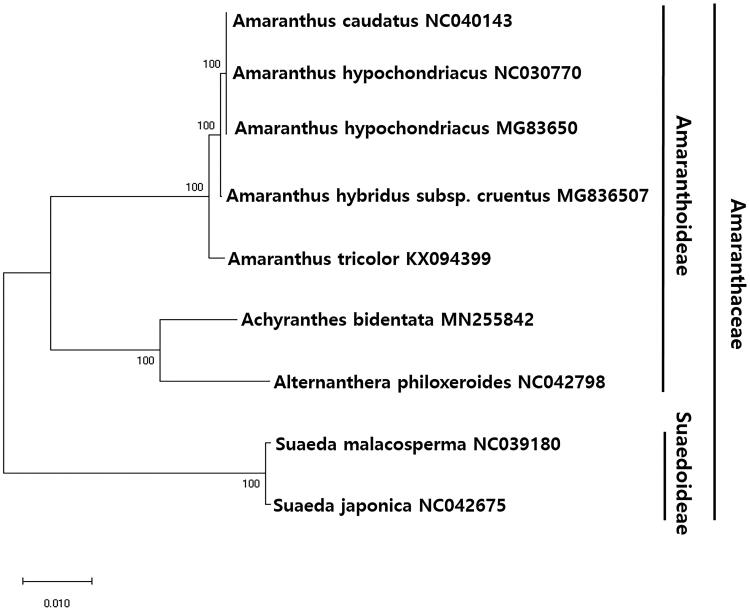
Maximum-likelihood (ML) tree based on the chloroplast protein-coding genes of 8 taxa, including *A. bidentata* and two outgroup taxon. Sixty-four protein-coding genes were aligned using MAFFT (Katoh et al. 2002). ML analysis was performed using MEGA6, with 1000 bootstrap replicates (Tamura et al. 2013). The bootstrap support values from 1000 replicates are indicated at the nodes.

## References

[CIT0001] Korea Institute of Oriental Medicine (KIOM) 2019 Defining dictionary for medicinal herbs. Available online: http://boncho.kiom.re.kr/codex/

[CIT0002] Moon BC, Choi G, Yuan Y 2017 Origins of herbal medicines and adulterants in Korea and China. Daejeon (South Korea): Korea Institute of Oriental Medicine.

[CIT0003] Kim K, Lee SC, Lee J, Lee HO, Joh HJ, Kim NH, Park HS, Yang TJ 2015 Comprehensive survey of genetic diversity in chloroplast genomes and 45s nrDNAs within *Panax ginseng* species. PLoS One. 10:e0117159. 26061692 10.1371/journal.pone.0117159 PMC4465672

[CIT0004] Park I, Kim WJ, Yang S, Yeo SM, Li H, Moon BC 2017 The complete chloroplast genome sequence of *Aconitum coreanum* and *Aconitum carmichaelii* and comparative analysis with other *Aconitum* species. PLoS One. 12:e0184257. 28863163 10.1371/journal.pone.0184257 PMC5581188

[CIT0005] Katoh K, Misawa K, Kuma KI, Miyata T 2002 MAFFT: a novel method for rapid multiple sequence alignment based on fast Fourier transform. Nucleic Acids Res. 30:3059–3066. 12136088 10.1093/nar/gkf436 PMC135756

[CIT0006] Tamura K, Stecher G, Peterson D, Filipski A, Kumar S 2013 MEGA6: Molecular Evolutionary Genetics Analysis version 6.0. Mol Biol Evol. 30:2725–2729. 24132122 10.1093/molbev/mst197 PMC3840312

